# The Japanese Association of Cardiovascular Intervention and Therapeutics position statement on coronary invasive procedures during the COVID-19 pandemic in Japan

**DOI:** 10.1007/s12928-021-00767-6

**Published:** 2021-03-22

**Authors:** Yuji Ikari, Shinichiro Yamada, Natsuhiko Ehara, Ken Kozuma, Toshiro Shinke, Teruyasu Sugano, Fumiyasu Seike, Shinjyo Sonoda, Junichi Tazaki, Takafumi Tsuji, Yoshiaki Mibiki, Takashi Muramatsu, Takashi Morita, Mitsuaki Sawano

**Affiliations:** 1 The Japanese Association of Cardiovascular Intervention and Therapeutics, Tokyo, Japan; 2 COVID-19 Task Force Subcommittee in Japanese Association of Cardiovascular Intervention and Therapeutics (CVIT), Tokyo, Japan

## Preface

The coronavirus disease 2019 (COVID-19) has become a global pandemic since the beginning of 2020 and has spread across regions in our nation as well. During the early stage of the pandemic, cardiology practices were forced to limit their quantity of interventional procedures largely due to (1) insufficient understanding of the nature of the disease, (2) inadequate health care system to cope with the risk of COVID-19 infection, and (3) lack of personal protective equipment (PPE) required to reduce the risk of infection for healthcare personnel [1]. In the absence of curative medical treatment, it is crucial for cardiology practices to build strategies that enable the performance of necessary cardiovascular procedures while minimizing the risk of contracting the virus. This position statement has been published to serve this purpose. Please note that this position statement does not intend to overrule already existing local guidelines and rules established by in-house Infection Control Teams and cardiovascular practitioners should follow them accordingly.

## Screening testing prior to diagnostic catheterization and coronary intervention

1. Patients undergoing elective procedures

All patients are recommended to undergo screening testing for COVID-19 [2, 3].

1. History taking
2. Symptoms: Symptoms that have occurred within 10 days; body temperature above 37.5℃, chills, cough, dyspnea, new-onset of change in taste or smell, myalgia, headaches, diarrhea, etc [4].
3. Contacts: Previous contacts with known COVID-19 patients in the past 10 days.
4. Travel history: Traveling overseas or to endemic areas in the past 10 days.
5. Imaging
6. Performance of plain chest CT scanning is useful for detecting severe cases of COVID-19; however, it does not hold a high sensitivity for detecting COVID-19-positive asymptomatic/mild cases. Thus, findings on the plain chest CT ought to be carefully interpreted and discussed with diagnostic radiologists when available.
7. PCR testing
8. Patients with a medium to high probability of COVID-19 infection based on the patient’s history and imaging studies are recommended to undergo PCR testing.
9. Patients with a low probability of COVID-19 infection based on the patient’s history and imaging studies are not recommended to undergo routine PCR testing since a certain rate of false negative results is expected among the low-risk population and it is considered not cost-effective. Nonetheless, if the treating hospital is situated in an endemic region and has the capacity to perform a sufficient number of PCR tests, the performance of PCR testing for low-risk, asymptomatic patients may be reasonable. The decision on whether or not to perform such tests is left to the discretion of each treating hospital.
10. Guidance for patients
11. Guide patients to limit their risk of infection two weeks prior to their planned procedure. Guidance can theoretically reduce the risk of asymptomatic patients to develop symptoms after admission.
12. If a definitive diagnosis of COVID-19 is made through the above-mentioned screening tests, it is recommended to postpone the planned cardiovascular procedure to a later time after active infection subsides.
13. If the PCR test is negative in a medium to high probability patient, it is recommended to postpone the planned cardiovascular procedure until COVID-19-related symptoms are not reported in the next 14 days or more.
14. If a definitive diagnosis of COVID-19 is made or the probability of active infection cannot be ruled out but the patient requires an urgent cardiovascular procedure, consider transferring the patient to a fully equipped hospital where cardiovascular procedures can safely be performed to COVID-19 patients.
15. Patients undergoing emergency procedures
16. History taking:
17. Refer to the previous section of patients undergoing elective procedures.
18. In cases of patients under great distress (congestive heart failure, cardiogenic shock, cardiac arrest, etc.), taking the complete history would not be feasible; thus, the patient should be regarded as a COVID-19-suspected case.
19. Imaging
20. In cardiovascular emergency cases, a CT scan is frequently performed. Routine CT scan is not recommended because of radiation exposure and low sensitivity for COVID-19 diagnosis. However, if a CT scan is performed, the lung area should also be evaluated as a possible target of COVID-19 infection.
21. PCR testing
22. If COVID-19 infection is in doubt based on history and imaging studies, we recommend performing PCR testing.
23. Qualitative antigen testing is an available alternative to PCR but is not recommended as a screening test since, although it provides the results within a short time, its sensitivity is 50–60 percent and has a considerable rate of false positives. In contrast, quantitative antigen testing is a better alternative to PCR with a sensitivity of 90 percent. The treating physician must be aware that all tests have different optimal timing depending on the individual’s stage of COVID-19 infection.

## Protective measures during elective procedures

As a general rule, elective procedures should be performed only for non-COVID-19 patients (SARS-CoV-2 negative). However, it is difficult to completely rule out COVID-19 in all patients in areas or at times of high alert because false negatives have been observed among those who underwent PCR testing. Therefore, it is necessary to prevent infection at the time of the procedure as well. The following are measures to minimize the risk of infection from asymptomatic or mild-symptom COVID-19 patients who may slip through initial screening tests. Individual facilities should determine whether or not the following measures are necessary depending on the level of infection [4].

1. Standard precaution for medical personnel
2. Surgical masks

All medical personnel should wear surgical masks in their facilities at all times.

(2) Eye protection with goggles or a face shield

The SARS CoV-2 virus enters the human body via the mucosal surface, and there is a possibility of infection through the eyes. If a cardiovascular patient is wearing a surgical mask correctly, protection for the eyes is generally unnecessary, but it is recommended that goggles or a face shield should be worn when dealing with a patient who is not wearing a surgical mask correctly or who may remove it during care.

2. Personal protective equipment in the catheterization laboratory
(1) Personal protective equipment for coronary intervention operators
1. PPE should be worn in the same way as instructed for surgical situations. In addition to the surgical gown and gloves, a surgical mask, cap, and goggles (or a face shield) must be worn.
2. The risk of infection is greatest during the undressing of PPE when gowns and gloves are contaminated with SARS CoV-2. Therefore, it is recommended that every healthcare worker in the catheterization lab be familiar with PPE dressing and undressing, and that double-checking during undressing be done by two or more persons. Particular attention should be paid to hand hygiene after PPE undressing by cleansing the operator’s hands with alcohol. The correct way to dress and undress PPE can be learned using online learning materials on domestic and foreign infectious diseases society websites.
(2) Personal protective equipment for coronary intervention assistants (nurses, medical engineers, etc.)

Always wear gloves, surgical mask, cap, goggles, or a face shield.

(3) Precautions toward the risk of infection from the patient

All patients undergoing procedures must always wear a surgical mask prior to transportation from their ward or upon entering the catheterization lab. Use a nasal cannula or an oxygen mask with a surgical mask if oxygen supply is required.

(4) Ventilation and sanitation of the catheterization lab after procedure

Routine ventilation and sanitation are not required after the procedure for low probability patients. However, if saliva or other body fluids have attached to the surface of the catheterization lab table, it should be wiped off for sanitation. If a high quantity of aerosol has been generated during a patient’s critical state, environmental ventilation and sanitation is required after the procedure.

3. Problems with our standard ventilation systems in the catheterization lab

Catheterization labs are generally designed to create a positive air pressure environment to reduce the risk of external pathogens entering the catheterization lab in accordance with current guidelines. Thus, they are not suitable for treating patients with a known or probable infection that can be transmitted via aerosols. The amount of time required for ventilation after procedures on these patients will vary from hospital to hospital and should be estimated as preparation for cases where a high quantity of aerosol emission is expected or has occurred during the procedure.

## Protective measures during emergency procedures

Since emergency procedures need to be pursued within a limited amount of time, preprocedural screening for COVID-19 can be challenging compared to elective procedures. It is important to note that COVID-19 itself can directly cause cardiovascular diseases such as acute coronary syndromes, pulmonary embolisms, and myocarditis. Therefore, patients manifesting sudden onset cardiovascular disease have a higher likelihood for COVID-19 infection; thus, it is difficult to discard the possibility of COVID-19 infection. Emergency procedures for patients with a definite or high probability of COVID-19 infection are recommended to be performed only at institutions with full equipment and personnel to deal with the COVID-19 infection [5, 6]. Emergency procedures can be performed similar to elective procedures under standard precaution if the patient has a low probability of infection.

1. Timing of the procedure
2. ST elevation myocardial infarction
3. When STEMI is suspected, perform an ECG and a TTE to confirm the diagnosis. Among COVID-19 patients, viral myocarditis can mimic STEMI on an ECG, making it difficult to differentiate between the two causes of ST elevation. In Japan, there has been a case where an emergency catheterization procedure was performed to a suspected STEMI case when in fact it was due to COVID-19 viral myocarditis. It is important to evaluate whether or not a segmental asynergy of the left ventricular wall is present on a TTE to confirm the diagnosis of STEMI.
4. Alongside the above-mentioned examination needed for STEMI diagnosis, perform a rapid screening test to determine the probability of COVID-19 infection via history taking, physical examination, imaging, and PCR testing in high probability cases. If the probability is deemed low, proceed with urgent catheterization to achieve prompt reperfusion. Performance of PCR testing is not necessary if the probability of COVID-19 infection is deemed low based on the patient’s history, physical examination, and imaging results. Procedures should be conducted in the same manner as those for elective procedures. Please note that a subtle delay in coronary intervention performance measures such as door-to-balloon time should be considered acceptable since screening and infection control measures against COVID-19 have been reported to take up extra time prior to the procedure.
5. If a definite or highly probable COVID-19 patient is treated at a COVID-19 hospital, early reperfusion therapy with coronary intervention should be considered. If a definite or highly probable COVID-19 patient is treated at a non-COVID-19 hospital, a transfer to a COVID-19 hospital should be considered if coronary intervention can be made within 60 min. If the transportation is not feasible within 60 min, or the patient’s hemodynamic status is rapidly deteriorating, reperfusion therapy with intravenous administration of tPA should be considered.
6. Non-ST elevation acute coronary syndrome
7. In a hemodynamically stable non-ST elevation ACS patient, we consider performing coronary intervention if there is a low probability of COVID-19 infection after sufficient screening tests have been performed. Procedures are recommended to be performed during times when full infection control measures are available (e.g., daytime for some facilities).
8. In a hemodynamically stable non-ST elevation ACS patient but with a high probability of COVID-19 infection upon presentation, PCR testing is recommended. If the PCR is negative and the risk of infection is deemed low, proceed with the planned procedure.
9. In a COVID-19-positive hemodynamically stable non-ST elevation ACS patient, supportive therapy for COVID-19 should be prioritized. The performance of coronary procedure after the risk of infection has minimized should be discussed depending on the patient’s status. Consider transferring the patient to a COVID-19 hospital if first treated at a non-COVID-19 hospital.
10. In a hemodynamically unstable non-ST elevation ACS patient, the performance of coronary intervention should be decided according to the STEMI patients.
11. Emergency procedures for patients with acute respiratory distress
12. Oxygen supplementation
13. Oxygen should be administered via a nasal cannula or an oxygen mask with a surgical mask covering. Healthcare providers should be aware of coughs that can generate aerosols within the catheterization lab.
14. Ventilators
15. Intubation for patients with severe respiratory distress should be performed in the emergency department or the intensive care unit capable of generating a negative air pressure environment with an N95 mask and full PPE before the procedure due to an expected high quantity of aerosol emission which entails a higher risk of infection for healthcare personnel. Intubation should be avoided whenever possible inside the catheterization labs since they are susceptible to infection, especially aerosol-mediated disease. Immediately after intubation, a closed-circuit ventilator with HEPA filters should be used. Use a HEPA filter between the intubation tube and the bag valve if temporary ventilation using a bag valve mask is needed before connection to a ventilator. A lower-than-standard management threshold for intubation is recommended to avoid the risk of intubation or use of non-invasive positive pressure ventilation in the catheterization lab upon the acute onset of respiratory distress. The type of ventilators and their ventilation circuit should be identified since not all ventilators have closed circuits.
16. NPPVs have been used widely for cardiac patients with acute respiratory distress due to their low grade of invasiveness. However, they can cause high-quantity aerosol emission when air leaks from NPPV masks, and large-scale clusters involving NPPV use have been reported previously. For these reasons, NPPVs are recommended not to be used during the COVID-19 pandemic. In a case when NPPVs must be used, healthcare personnel should wear PPE with N95 masks, and the use of NPPVs should be limited within a negative air pressure environment outside the catheterization lab.

## Infection control for definite or highly probable COVID-19 patients in the catheterization lab

As stated earlier, definite or highly probable COVID-19 patients are highly advised to be treated only at COVID-19 hospitals even in the case of ACS. The following are the recommended infection control measures that should be taken during care for definite or highly probable COVID-19 patients in the catheterization lab.

1. Minimizing the risk of healthcare personnel infection

During a procedure for definite or highly probable COVID-19 patients, the number of healthcare personnel (interventionalist, nurse, medical engineer, radiology technician, etc.) should be limited to the least number necessary for the procedure to minimize the risk of healthcare personnel infection. For example, when more than one interventionalist is available within the facility, limit the interventionalist to one without an assistant during a procedure.

2. PPE within the catheterization lab

All healthcare personnel within the catheterization lab are required to cover themselves with full PPE. Full PPE including an N95 mask, a face shield or goggles, a cap, a gown, and gloves (preferably two pairs of gloves) is mandatory during the procedure. A fit test should be performed prior to wearing an N95 mask, and a seal check must be performed after an N95 mask is worn prior to the procedure.

3. Preparation of the catheterization lab

Remove all the equipment unnecessary for the procedure from the catheterization lab to reduce the risk of infection whenever possible. The major infection route of COVID-19 is via droplet contact infection. The use of disposable sheets under the treated patient may be effective to reduce the risk of infection from high concentration contamination in the catheterization lab. Procedures for non-ST elevation myocardial infarction ACS patients are recommended to be performed at the end of the day after all elective procedures, if possible. After a procedure, negative air pressure ventilation, decontamination using either sodium hypochlorite, potassium peroxoacid sulfate-soaked cloth, or 70 degrees or greater ethanol is mandatory for the purpose of terminal cleaning. A short duration of ultraviolet radiation is an alternative method for decontamination. If a certain amount of ventilation can be ensured, the catheterization lab can be used for the next patient after dedicating appropriate time for ventilation and environmental decontamination. The optimal stoppage time for ventilation should be calculated a priori depending on the catheterization lab’s ventilation capability. Generally, catheterization labs are designed as level III clean areas that require approximately one hour of ventilation until enough aerosols are removed from the air. If there are more than one catheterization labs within a facility, the catheterization lab to be used for definite or high probability COVID-19 cases should be specified.

## Cooperation between hospitals during the COVID-19 pandemic

During the pandemic, there is a high probability that patients requiring urgent medical procedures will be delivered to hospitals without sufficient protection against COVID-19. Therefore, a priori partnership agreements between COVID-19 hospitals and non-COVID-19 hospitals would be crucial for delivering urgent care to these patients, particularly for non-COVID-19 hospitals.

## When the pandemic worsens in Japan

Daily increase in the number of COVID-19-infected patients is continually reported as of December 2020. We cannot deny the fact that situations may worsen to the extent that we have observed in the United States and European countries. In the case of a lockdown where the local authority or the government puts restrictions on daily activities of businesses unrelated to basic social infrastructure (e.g., police, fire department, construction, manufacturing, food, pharmacies, or other stores that sell everyday goods), elective procedures are recommended to be postponed to a later time if the patient is in such a condition. Elective procedures are recommended to be postponed to a time when the conditional re-opening of small businesses unrelated to basic social infrastructure or parks is allowed. Nonetheless, the timing of stopping or restarting elective procedures should be thoroughly discussed within each facility, since there is a high demand for human resources during a pandemic.

## Concluding Remarks

We are beginning to see a path toward a solution with the accelerating amount of research on SARS CoV-2, treatment, and vaccine development against COVID-19. However, the effect of the pandemic is expected to stay for the time being, and may even worsen during the coming months. To maintain the usual care for cardiovascular patients, infection control cannot be ignored. We wish that hospitals all around Japan would refer to this statement and create an in-house infection control guideline customized to their local needs to protect their healthcare personnel and continue usual care for their patients.
